# Research Progress on the Alkaloids of *Dendrobium nobile*: Substantiation, Key Components, Pharmacological Activity, and Biosynthetic Pathways

**DOI:** 10.3390/cimb48060570

**Published:** 2026-05-29

**Authors:** Changli Yang, Qi Jia, Yongxia Zhao, Lin Qin, Daopeng Tan, Yuqi He

**Affiliations:** 1 Guizhou Engineering Research Center of Industrial Key-Technology for *Dendrobium nobile*, Guizhou Engineering Research Center for Orchid Medicinal Plant Breeding and Efficient Application, Zunyi Medical University, Zunyi 563000, China; 15121420521@163.com (C.Y.); jiaqi@zmu.edu.cn (Q.J.); x.y.z.100@163.com (Y.Z.); qinlin1115@163.com (L.Q.); 2 Bioresource Institute for Healthy Utilization, Zunyi Medical University, Zunyi 563009, China

**Keywords:** *Dendrobium nobile*, dendrobine, alkaloids, biosynthetic pathway, pharmacological action

## Abstract

*Dendrobium nobile* Lindl. is a valuable medicinal orchid in traditional Chinese medicine. It abounds in alkaloids with extensive pharmacological properties, holding promising prospects for pharmaceutical development. In this review, the relevant literature was systematically retrieved from PubMed, Web of Science and CNKI using the keywords *Dendrobium nobile* Lindl., alkaloids, dendrobine, pharmacological activities and biosynthetic pathways. We comprehensively summarize the chemical composition, pharmacological effects, biosynthetic routes, clinical application potential and research prospects of alkaloids derived from *D. nobile*. The main alkaloid components including dendrobine, dendramine and nobilonine are classified into dendrobine, dendroxine and nobiline structural types. This paper further elaborates the anti-inflammatory, antioxidant and neuroprotective effects of these alkaloids, as well as their regulatory mechanisms on apoptosis-associated proteins and signaling cascades. Key enzymes and regulatory genes participating in the mevalonate pathway-mediated biosynthesis of sesquiterpenoid alkaloids are also discussed. Collectively, this review provides a theoretical basis and reference for subsequent basic research and therapeutic development of *D. nobile* alkaloids.

## 1. Introduction

Globally, there are roughly 1000 *Dendrobium* species, more than 50 of which possess medicinal value. Representative medicinal species include *Dendrobium candidum* Wall., *Dendrobium huoshanense* and *Dendrobium nobile* Lindl. (*D. nobile*). As a perennial epiphytic herb of the Orchidaceae family, *D. nobile* is most famously represented by the germplasm native to Chishui, Guizhou Province. Boasting exquisite and graceful blossoms, it is widely cultivated as an indoor ornamental flower. Meanwhile, it serves as a classic traditional Chinese medicinal material, with fresh and dried stems acting as the major medicinal parts, while flowers, leaves and roots are also used secondarily [1].

*D. nobile* accumulates diverse bioactive constituents such as alkaloids, polysaccharides, sesquiterpenes and bibenzyl compounds. In recent decades, alkaloids and polysaccharides have drawn intensive research interest owing to their broad therapeutic effects. Dendrobine, stipulated as the quality evaluation indicator of *D. nobile* in the 2020 edition of Pharmacopoeia of the People’s Republic of China, exerts prominent analgesic, antipyretic, antiviral, hypoglycemic and anti-inflammatory effects, and thus gains widespread clinical attention.

Nevertheless, the clinical translation and pharmaceutical development of dendrobine remain inadequate, largely owing to its scarce natural supply. Currently, dendrobine is mainly acquired through three approaches: direct extraction from *D. nobile*, chemical synthesis and microbial biosynthesis. Wild *D. nobile* is an endangered medicinal plant and fails to satisfy market supply demands. Moreover, the intricate molecular structure of dendrobine creates technical hurdles. Existing chemical synthetic routes suffer from harsh reaction conditions and low product yields, greatly restricting their practical utilization [2]. Accordingly, clarifying the biosynthetic mechanism of dendrobine is essential to improve its production yield and lay a solid theoretical basis for its pharmaceutical exploitation.

## 2. Alkaloids

**Figure 1:**
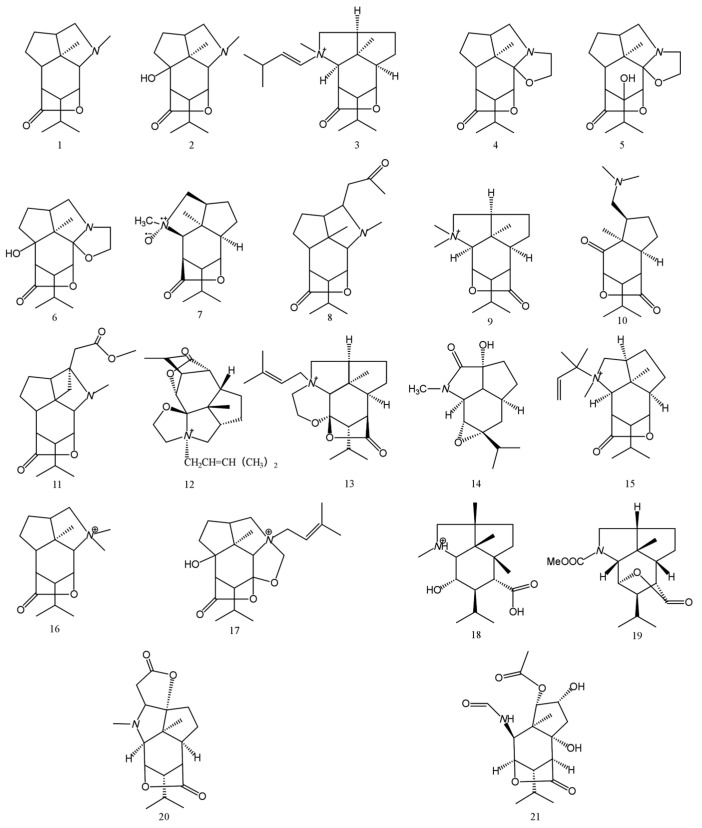
Structure of Dendrobium-type alkaloids isolated from *D. nobile*.

Alkaloids represent a structurally diverse and complex class of natural products, derived from *Dendrobium* sp. can be categorized into five principal types: sesquiterpenes, octahydroindoles, tetrahydropyridines, amides, and imidazoles. Among these, sesquiterpene alkaloids are further subdivided into four subtypes: dendrobine-type, dendroxine-type, nobiline-type, and others [3]. The fundamental structure of dendrobine-type alkaloids consists of a pyrrolidine-integrated sesquiterpene scaffold, characterized by an embedded five-membered C_2_-C_9_ heterocycle containing nitrogen and a fused C_3_-C_5_ lactone ring system. To date, 21 dendrobine-type alkaloids have been isolated from different tissues of *D. nobile* (Figure 1, Table 1). Notably, six major alkaloids-including dendrobine, dendramine, and N-isopentenyldendrobinium are distributed across flowers, stems, and leaves (compounds **1**–**8**), whereas the remaining alkaloids are predominantly stem-specific Spatial analysis of mature stems reveals distinct tissue-specific accumulation patterns, N-Methyl-dendrobinium is primarily localized in the cuticle layer. Dendrobine accumulates predominantly in the epidermis and vascular bundles but is minimally detected in parenchyma tissues, suggesting biosynthesis likely initiates in vascular and parenchymal cells followed by translocation to the epidermis during stem maturation. N-Isopentenyl-dendrobinium is concentrated in vascular bundles and parenchyma, whereas dendramine and dendrobine-N-oxide exhibit broad distribution across all stem tissues [4].

**Table 1:** Dendrobium-type alkaloids isolated from *D. nobile*.

Number	Name of the Compound	Molecular Formula	Location	References
1	Dendrobine	C_16_H_25_NO_2_	Stems, leaves and Flowers	[5,6]
2	Dendramine	C_16_H_25_NO_3_	Stems, leaves and Flowers	[7,8]
3	N-isopentenyldendrobinium	C_21_H_34_NO_2_^+^	Stems, leaves and Flowers	[9]
4	Dendroxine	C_17_H_25_NO_3_	Stems, leaves and Flowers	[10]
5	4-hydroxy-dendroxine	C_17_H_25_NO_4_	Stems, leaves and Flowers	[11]
6	6-hydroxy-dendroxine	C_17_H_25_NO_4_	Stems, leaves and Flowers	[7,12]
7	dendrobine-N-oxide	C_14_H_21_N_3_O_3_	Stems, leaves and Flowers	[10]
8	dendronobiline A	C_19_H_29_NO_3_	Stems, leaves and Flowers	[13]
9	N-methyldendrobinium	C_17_H_28_NO_2_^+^	Stems	[9]
10	nobiloline	C_17_H_27_NO_3_	Stems	[14]
11	dendrine	C_19_H_29_NO_4_	Stems	[15]
12	N-isopentenyl-dendroxine	C_22_H_34_NO_3_^+^	Stems	[10]
13	N-isopentenyldendrobinium	C_21_H_34_NO_2_^+^	Stems	[9]
14	3-hydroxy-2-oxodendrobine	C_16_H_24_NO_4_	Stems	[16]
15	N-isopentenyl-dendrobine	C_21_H_34_NO_2_^+^	---	[10]
16	N-methyl-dendrobine	C_17_H_28_NO_2_^+^	---	[8]
17	N-isopentenyl-6-hydroxydendroxine	C_22_H_34_NO_4_^+^	Stems	[5]
18	dendronboic acid	---	Stems	[17]
19	N-methoxylcarbonyldendrobine	---	---	[17]
20	dendroterpene F	C_18_H_25_NO_4_	Stems	[18]
21	dendroterpene F	C_17_H_25_NO_6_	Stems	[18]

## 3. Dendrobine

Dendrobine, a sesquiterpene alkaloid, is recognized as the characteristic quality marker for *D. nobile* in the Chinese Pharmacopoeia. Modern pharmacological studies have revealed its multi-target bioactivities, including gastrointestinal function modulation, neuroprotection, anti-inflammatory effects, and anti-influenza properties. Despite promising medicinal prospects, the shortage of natural resources severely hinders drug development and large-scale clinical application. At present, dendrobine is mainly acquired via three approaches.

### 3.1. Direct Extraction from D. nobile

Dendrobine was first isolated from wild *D. nobile*. Excessive wild harvesting, together with strict ecological adaptability and slow growth rate of this species, has caused a sharp decline in wild resources. Being an endangered plant, wild *D. nobile* can only produce low yields of dendrobine. Moreover, its alkaloid content varies greatly depending on cultivation patterns, altitude, plant tissues, harvesting years and stem segments. Such unstable content obstructs stable raw material supply for scientific research and industrial production [19]. Therefore, exploring the biosynthetic pathways and pivotal functional enzymes governing dendrobine synthesis is essential to address the bottlenecks caused by long growth cycles and environmental susceptibility.

### 3.2. Chemical Synthesis

Dendrobine was the first isolated from *D. nobile* by Suzuki et al. in 1932 [20], and its independent isolation and identification were subsequently accomplished by the research group of K. K. Chen in 1935 [21]. Owing to its intricate polycyclic fused architecture with multiple adjacent chiral centers, the chemical synthesis of dendrobine has remained a formidable challenge.

In 1970, Yamamoto’s group constructed the 6/6/5 tricyclic core skeleton of dendrobine through intermolecular Diels–Alder reaction, with racemic carvone derivative and 1,3-butadiene as raw materials. Based on this achievement, Kende et al. realized the first total synthesis of racemic dendrobine in 1979 [22].

**Figure 2:**
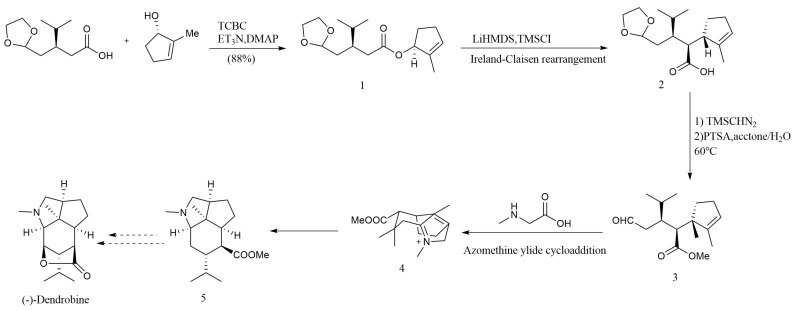
Synthetic study of (−)-dendrobine [26].

Corey’s team completed the formal synthesis of (−)-dendrobine in 2004, adopting chiral oxazaborolidinium catalyst to facilitate asymmetric Diels–Alder reaction [23]. In 2017, Chen and coworkers established a one-pot method to build the tetrahydropyrrolopentane carbon framework and further synthesized racemic dendrobine [24]. Despite multiple reported asymmetric total synthetic routes toward (−)-dendrobine, these protocols generally involve cumbersome reaction steps and costly raw materials or catalysts, which restrict their scaled-up practical production. A typical synthetic route proposed by Trauner’s group in 2018 is presented in Figure 2. This synthesis adopts Ireland–Claisen rearrangement to set critical chiral centers, and the azomethine ylide [3+2] cycloaddition serves as the key reaction for assembling the core 6/5/5 tricyclic structure [25,26]. Despite these elegant synthetic advances, chemical synthesis currently remains impractical for addressing the limited availability of dendrobine.

### 3.3. Microbial Synthesis

Endophytic fungi inhabiting *Dendrobium* exert vital effects on plant growth, stress tolerance and secondary metabolism regulation [27,28]. For instance, *Mycena* sp. MF23 regulates dendrobine accumulation via modulating the mevalonate (MVA) pathway [29]. *Trichoderma longibrachiatum* MD33 derived from *D. nobile* stems can synthesize dendrobine and participate in its biosynthetic process [30]. Recent evidence reveals that endophytic microbial communities differ across epiphytic habitats, thereby exerting divergent impacts on metabolite biosynthesis. The strain *Phyllosticta fallopiae* DN14 isolated from wild *D. nobile* leaves is positively associated with increased dendrobine content [31]. Nevertheless, current studies on *Dendrobium* endophytes mainly concentrate on species classification and stress resistance mechanisms, while their potential for large-scale microbial biosynthesis remains underexplored.

## 4. Biosynthesis of Dendrobine

**Figure 3:**
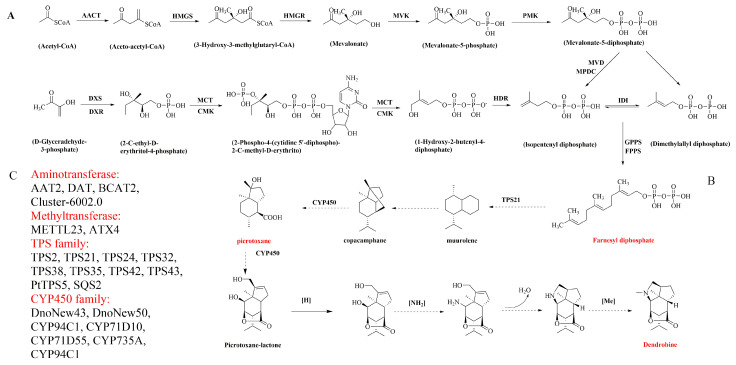
(**A**) Upstream pathway of dendrobine synthesis, depicting the MVA and MEP pathways that generate the C_5_ precursors IPP and DMAPP, which are subsequently condensed to form FPP. (**B**) Downstream pathway of dendrobine synthesis, illustrating potential skeletal structures and hypothesized intermediates derived from FPP via cyclization, oxidation, amination and methylation to produce dendrobine and related alkaloids. (**C**) Candidate genes involved in skeleton formation and post-modification were identified from genomic and transcriptomic data of *D. nobile*, including TPSs, cytochrome P450s, aminotransferases, methyltransferases and transcription factors, all of which participate in this biosynthetic process [36].

Dendrobine, a sesquiterpenoid alkaloid characterized by a picrotoxane-type skeleton, was the first alkaloid isolated from *D. nobile*. Its biosynthesis primarily proceeds through three core stages: precursor supply, skeleton formation, and structural modification [32]. According to the biosynthetic pathways of sesquiterpenoids, both the mevalonate (MVA) and methyl-D-erythritol 4-phosphate (MEP) pathways can generate the universal C_5_ precursors isopentenyl diphosphate (IPP) and dimethylallyl diphosphate (DMAPP). These intermediates are subsequently condensed by farnesyl diphosphate synthase (FPPS) to form the sesquiterpene backbone precursor farnesyl diphosphate (FPP). This catalytic step, which represents the committed entry point into sesquiterpenoid biosynthesis, is regarded as the initiation of the downstream pathway for dendrobine production [33] (Figure 3A).

Initially, farnesyl pyrophosphate (FPP) is catalyzed by terpene synthases (TPS) and cytochrome P450 oxidoreductases to produce skeleton A, which is further oxidized to form intermediate B. Intermediates C and D are successively generated via ring-opening, hydrolysis, and oxidation reactions. Intermediate D is reduced into intermediate E, and subsequent esterification yields intermediate F. Intermediate F enters two parallel biosynthetic branches. In the first branch, cyclization of F generates dendronobilin C, which undergoes amination to produce mubironine B and further methylation to synthesize dendrobine. In the second branch, F is directly aminated to form nobilonine, which is converted into dendrobine through cyclization and decarboxylation. Additionally, mubironine B can be transformed into the newly identified alkaloid dendroterpene F via a cascade of reactions, including transamination, oxidation, cyclization, methyltransferase-catalyzed methylation, dehydration and acetylation. Apart from the above pathways, intermediate E can also form a dendrobine-lactone precursor. The precursor is subjected to oxidation, amination and methylation, ultimately yielding dendrobine. Benefiting from the progress of multi-omics techniques, a large number of candidate genes involved in these catalytic processes have been successfully screened (Figure 3B).

Xu et al. performed genomic sequencing and obtained a 1.19 Gb genome assembly of *D. nobile* Lindl., annotating 29,476 protein-coding genes. Combined transcriptomic and genomic analyses showed that HMGS and MVD, two pivotal enzymes of the mevalonate (MVA) pathway, displayed markedly higher expression in stems than in other tissues [34]. Another whole-genome study of *D. nobile* generated a 1.19 Gb genome assembly with 31,672 predicted protein-coding genes, supporting the screening of candidate genes associated with picrotoxane-type sesquiterpenoid alkaloid biosynthesis. The key functional enzymes cover farnesyl diphosphate synthase (FPPS), malonyl-CoA:ACP transacylase (MCT), malonyl-CoA synthetase (MCS), 1-hydroxy-2-methyl-2-(E)-butenyl-4-diphosphate reductase (HDR), 4-diphosphocytidyl-2-C-methyl-D-erythritol kinase (CMK), mevalonate diphosphate decarboxylase (MVD), acetoacetyl-CoA transferase (AACT) and isopentenyl diphosphate isomerase (IDI). Given the dominant expression pattern of MEP pathway-related genes, dendrobine biosynthesis is inferred to mainly rely on the methyl-D-erythritol 4-phosphate (MEP) pathway [35].

In the downstream pathway of dendrobine biosynthesis, multiple cytochrome P450 monooxygenases (CYP450s) mediate oxidation and hydroxylation reactions in *Dendrobium* species and their endophytic fungi, significantly enhancing the structural diversity of dendrobine-type sesquiterpenoid alkaloids [36] (Figure 3C). Studies indicate that DnoNew43 and DnoNew50 may function analogously to CYP72A1, serving as candidate genes involved in dendrobine biosynthesis. Additionally, terpene synthase (TPS) acts as a key enzymatic gatekeeper in terpenoid biosynthesis, catalyzing the transformation of acyclic prenyl diphosphate precursors into diverse hydrocarbon scaffolds [37]. The terpene synthase (TPS) family is classified into five major subfamilies: TPS-a, TPS-b, TPS-c, TPS-e/f, TPS-g, and TPS-h, with the TPS-b subfamily further subdivided into TPS-b-I, TPS-b-II, and TPS-b-III clades. *D. nobile* exhibits significantly higher dendrobine content compared to other *Dendrobium* species, a distinction correlated with the elevated expression of TPS-a genes in its stems, as confirmed by transcriptomic analyses. Subsequent investigations revealed that TPS-b-II and TPS-b-III isoforms may contribute to interspecific variation in dendrobine accumulation among *Dendrobium* taxa, potentially through differential regulation of sesquiterpenoid precursor flux [34].

Genomic and transcriptomic analyses have identified multiple genes within the TPS-a subfamily that contribute to the formation of the sesquiterpenoid backbone of dendrobine. Eight key TPS genes, including TPS2, TPS24, TPS35, TPS32, TPS38, TPS42, and TPS43, exhibit distinct tissue-specific expression patterns, with predominant expression in stems and floral tissues, which is consistent with the spatial accumulation characteristics of dendrobine. Comparative transcriptomic profiling further validates that these genes are significantly upregulated in dendrobine-enriched tissues, supporting their direct roles in catalyzing the cyclization of farnesyl pyrophosphate (FPP) to generate picrotoxane-type biosynthetic intermediates. Moreover, TPS21 has been functionally associated with dendrobine biosynthesis, presumably by producing sesquiterpene precursors such as germacrene D and β-caryophyllene, which act as essential substrates for downstream oxidation and amination reactions [34,38]. The sesquiterpene synthase PtTPS5 catalyzes the cyclization of farnesyl pyrophosphate (FPP) to yield two guaiane-type sesquiterpenoids: (1S,5S,7R,10R)-guaia-4(15)-en-11-ol and (1S,7R,10R)-guaia-4-en-11-ol [39]. Aminotransferases and methyltransferases are essential catalytic enzymes for the post-modification of plant secondary metabolites. Transcriptomic profiling of *D. nobile* colonized by the mycorrhizal fungus MF23 revealed that the core modifying enzymes responsible for dendrobine biosynthesis include the aminotransferases AAT2, DAT, and BCAT2 [40]. In addition, the glutamate-1-semialdehyde aminotransferase encoded by Cluster-6002.0 is considered a critical regulator of the transamination reactions required for dendrobine formation [41].

Methyltransferases (MTs) are widely distributed in plants and can be categorized into O-methyltransferases (O-MTs), C-methyltransferases (C-MTs), and N-methyltransferases (N-MTs) according to the functional groups of substrate atoms (i.e., oxygen, carbon, and nitrogen). Among these subclasses, N-MTs serve as key regulatory enzymes in purine alkaloid biosynthesis and mediate the conversion of xanthosine to theobromine and caffeine. The transcriptional abundance of N-methyltransferase genes is strongly and positively correlated with caffeine production [42]. To date, a total of five N-methyltransferase genes have been functionally verified to participate in dendrobine biosynthesis [35].

Numerous studies have confirmed that transcription factors (TFs), including the C3H, bHLH, bZIP, MYB, WRKY, and AP2/ERF families, serve as central regulators of genes involved in dendrobine biosynthesis [43,44]. These TFs precisely modulate the activity of pathway enzymes, thereby orchestrating the spatiotemporal accumulation of specialized metabolites such as terpenoids, alkaloids, and phenolic acids. For example, G2-like TFs are negatively correlated with terpenoid accumulation, whereas GRF TFs exert an opposite regulatory effect on alkaloid production. The DhbHLH TF exhibits context-dependent dual functions, acting as both a positive and negative regulator of alkaloid biosynthesis [45]. Additionally, DcMYB61 positively promotes dendrobine synthesis, while ERF, NAC, and MYB TFs contribute to terpenoid backbone biosynthesis in floral tissues. Li et al. performed weighted gene co-expression network analysis (WGCNA) and identified ten core TFs, namely ATHB-13, ATHB-13-1, MADS16, MADS16-1, GT-1, IPN2, MYB30, MYB101, and ERF109, which are presumed to modulate sesquiterpenoid skeleton assembly [35]. In another WGCNA-based study, Zhao et al. screened the key TF MYB61, and functional verification demonstrated that its overexpression increases dendrobine content by more than two-fold, significantly enhancing dendrobine biosynthesis [46]. These TFs likely facilitate dendrobine accumulation by coordinately activating core biosynthetic genes. Gong et al. further conducted co-expression analysis and identified 238 TFs associated with the activity of sesquiterpene synthase SQS2. Among them, TFs from the ZAT11, NAC, MYB, ZIM, HD-ZIP, and bHLH families showed positive correlations with SQS2 activity, whereas B3, WRKY, CCCH, EREBP-1, BEL1, GRF6, and BTB/POZ family members exhibited negative correlations. These divergent regulatory patterns collectively shape terpenoid diversity and modulate dendrobine biosynthesis [29].

To fully validate the complete dendrobine biosynthetic pathway, the construction of an intermediate compound library, combined with dynamic monitoring of dendrobine accumulation, enables the targeted isolation of key intermediates at critical biosynthetic stages. Advances in multi-omics technologies have greatly accelerated the mining of candidate genes associated with dendrobine biosynthesis. Genomic studies have annotated 29,476 to 31,672 protein-coding genes in *D. nobile*, and the upregulated expression of MVA pathway genes in stems is closely associated with alkaloid accumulation. Transcriptomic evidence further reveals that terpene synthase and cytochrome P450 genes display tissue-specific expression in vascular bundles and the epidermis, which is consistent with the spatial distribution pattern of dendrobine.

Nevertheless, functional validation of these candidate genes remains insufficient, largely due to the lack of efficient genetic transformation systems for *D. nobile*. Future research should focus on integrated strategies to resolve this bottleneck, including the following approaches: RNA interference (RNAi) and CRISPR/Cas9-mediated gene knockout to silence key candidate genes and characterize their effects on metabolic intermediate profiles; heterologous expression in microbial chassis to reconstruct modular biosynthetic pathways and optimize enzymatic activity; and host metabolic engineering via promoter modification and TF co-expression to enhance metabolic flux toward dendrobine synthesis. The synergistic combination of these technical tools, together with advanced delivery systems and multi-omics integrative analysis, will contribute to fully elucidating the regulatory mechanisms underlying dendrobine biosynthesis and promoting its sustainable microbial production.

## 5. Pharmacological Effects of Dendrobine

### 5.1. Anti-Inflammatory Effects

Alkaloids extracted from *D. nobile* possess prominent anti-inflammatory properties mediated by multiple molecular pathways. These compounds exert broad-spectrum anti-inflammatory, anti-fibrotic, and tissue-protective activities through a multi-layered and multi-target signaling network. Specifically, dendrobine inhibits reactive oxygen species (ROS) accumulation and blocks the p38–c-Fos/NFATc1 signaling axis, thereby downregulating matrix metalloproteinase-9 (MMP-9) expression. It effectively suppresses osteoclast formation in vitro and ameliorates inflammatory osteolysis in vivo, making it a promising candidate agent for the treatment of bone erosion-associated disorders [47].

Other *D. nobile* alkaloids also exhibit potent anti-inflammatory bioactivity. Xu et al. isolated dendronobilonine A and nobiline from *D. nobile*, both of which significantly inhibit lipopolysaccharide (LPS)-induced nitric oxide (NO) production in RAW264.7 macrophages, with IC_50_ values of 16.7 μM and 24.4 μM, respectively [48]. In addition, *D. nobile* total alkaloids (DNLA) alleviate neuroinflammation by restraining the excessive activation of astrocytes and macrophages via modulation of the p38 MAPK/NF-κB pathway, which reduces the secretion of pro-inflammatory cytokines, including TNF-α, IL-6, and IL-1β [48].

Accumulating evidence has revealed that the anti-inflammatory actions of dendrobine exhibit high consistency and distinct tissue specificity. In thyroid-associated ophthalmopathy (TAO), dendrobine targets AKT1 and suppresses the AKT/NF-κB cascade, thereby mitigating ocular inflammation and fibrosis [49]. In neuroinflammatory contexts, dendrobine reduces ROS generation, inhibits the upstream transcription factor Runx1, and subsequently represses NF-κB activation, while modulating microglial M1/M2 polarization to achieve neuroprotection. In inflammatory bone diseases, however, dendrobine preferentially functions through the p38/c-Fos/NFATc1 pathway in an NF-κB-independent manner [50]. Importantly, these alkaloids exert anti-inflammatory effects at effective doses without obvious cytotoxicity, demonstrating their selective pharmacological activity. Collectively, ROS act as conserved upstream regulators responsible for the anti-inflammatory efficacy of dendrobine. Key kinases (p38 and AKT) and transcription factors (NF-κB, NFATc1, and Runx1) constitute a central signaling network that synergistically mediates the comprehensive anti-inflammatory responses of *D. nobile* alkaloids.

### 5.2. Anti-Aging Effects

Currently, research on the anti-aging effects of dendrobine has covered three different levels of models—yeast, in vitro cells, and mammals—forming a complete chain of evidence from lower organisms to higher animals, with clear mechanistic interconnections across the models.

In yeast models, dendrobine extends lifespan by regulating the Sch9/Rim15/Msn2 longevity-related pathway, scavenging ROS, enhancing antioxidant enzyme activity, and activating autophagy [51]. This finding provides an upstream evolutionary starting point for understanding the anti-aging mechanism of dendrobine: Sch9 is a functional homolog of the mammalian mTOR/S6K and AKT pathways, while Rim15/Msn2 is functionally similar to mammalian FOXO transcription factors.

In in vitro cell experiments, the mechanism of dendrobine is highly consistent with that observed in yeast models. It acts via the Nrf2/Keap1 and SIRT1/FOXO3a signaling axes to inhibit oxidative stress- and inflammation-induced cellular senescence, reduce the levels of senescence markers, and prevent the release of the senescence-associated secretory phenotype [52]. Notably, the SIRT1/FOXO3a pathway represents the evolutionary continuation of the yeast Rim15/Msn2 signaling axis in mammalian cells, while the Nrf2-mediated antioxidant response reflects the cellular-level regulation of oxidative stress downstream of the Sch9 pathway.

In mammalian aging models, the mechanisms of dendrobine and DNLA from *D. nobile* further integrate the core findings from the previous two model layers. At the gene expression level, they downregulate damage-related genes (e.g., Lmtk3, Usp10, Dzip1) and upregulate neuroprotective factors (e.g., Kctd16, Psd3, Bsn), effectively ameliorating neuronal damage and cognitive decline in aged mice [53]. At the signaling pathway level, dendrobine alleviates cellular senescence in multiple tissues via the ROS/NF-κB and mitophagy pathways. Mitophagy regulation is directly coupled with activation of the SIRT1/FOXO3a pathway, while ROS/NF-κB inhibition is closely linked to the Nrf2-mediated antioxidant effect. The mutual corroboration among these models provides a solid theoretical foundation for interventions in aging-related chronic diseases, functional product development, and candidate drug discovery.

### 5.3. Anti-Diabetic Effects

DNLA exert prominent anti-diabetic effects by modulating glucose metabolism and insulin signaling pathways. In alloxan-induced diabetic rats, DNLA reduces blood glucose levels through a multi-target regulatory mechanism. Subsequent studies have demonstrated that DNLA upregulates hepatic IRS-2 and IGF-1 expression, enhances insulin sensitivity, and ameliorates insulin resistance under high-fat and high-sugar dietary conditions. Although IRS-1 and IRS-2 share high structural homology, they mediate distinct biological functions within the insulin/IGF-1 signaling cascade. Rabiee et al. reported that IRS-1 knockout markedly diminishes global protein phosphorylation in response to IGF-1 stimulation, whereas IRS-2 knockout triggers more specific signaling dysfunction, indicating that IRS-1 and IRS-2 perform indispensable and non-redundant functions in insulin/IGF-1 signal transduction [54]. Furthermore, in a genetically modified mouse model of gestational diabetes mellitus (GDM), oral administration of 20 mg/kg dendrobine significantly decreased maternal blood glucose, corrected aberrant gestational weight gain, and relieved GDM-associated adverse outcomes, including fetal macrosomia [55]. Mechanistically, dendrobine may suppress the proliferation and differentiation of Th17 cells, reduce the abundance of circulating pro-inflammatory cytokines, and protect insulin signaling from inflammation-mediated impairment. By alleviating chronic low-grade inflammation-induced metabolic disorders, dendrobine improves glucose homeostasis and insulin resistance. Nevertheless, several critical issues remain to be systematically explored, including the long-term gestational safety of dendrobine, human equivalent safe doses, placental permeability, and its persistent influences on offspring development, which require further preclinical investigation and clinical validation.

### 5.4. Anti-Tumor Activity

Studies demonstrate that DNLA and dendrobine inhibit breast cancer cell apoptosis and suppress lung cancer progression. Li proposed that dendrobine suppresses the PD-1/PD-L1 signaling pathway, upregulates caspase-3 expression, and downregulates Ki-67 expression to restrict lung tumor growth. Although cisplatin chemotherapy is a primary anticancer strategy, its efficacy is limited by cardiotoxicity and other side effects. Recent studies indicate that combining dendrobine with cisplatin not only mitigates cisplatin-induced toxicity but also enhances antitumor efficacy [56]. Song revealed that dendrobine induces apoptosis via a mitochondrial-mediated pathway, activating the JNK/p38 stress signaling cascade, upregulating pro-apoptotic proteins Bax and Bim, and ultimately triggering cell death. Additionally, dendrobine significantly alleviates cisplatin-induced weight loss and cardiotoxicity in nude mice, highlighting its potential in managing chemotherapy-related adverse effects [57]. Luo investigated the combined effect of dendrobine and cisplatin on non-small cell lung cancer (NSCLC). In vivo experiments showed that the combination extended survival and reduced tumor volume in H1299 cell-xenografted mice [58].

Notably, in vitro experiments showed that neither single use of dendrobine nor its combination with cisplatin significantly affected the clonogenic capacity or apoptosis level of H1299 cells. This obvious difference between in vivo and in vitro effects suggests that dendrobine does not exert its therapeutic effect mainly through direct cytotoxicity or induction of apoptosis on tumor cells; instead, its therapeutic effect is more likely mediated by the tumor microenvironment, especially through immune regulation.

Further mechanistic studies have confirmed that dendrobine can regulate the immune microenvironment by modulating the balance between regulatory T cells (Treg) and T helper 17 cells (Th17). Specifically, dendrobine can inhibit Treg differentiation, downregulate Foxp3 expression, enhance Th17 cell activity, and increase serum IL-17 levels. These findings provide direct experimental evidence for the immune regulatory mechanism of dendrobine. Therefore, the synergistic anti-tumor effect of dendrobine combined with cisplatin is mainly derived from its immune regulation by restoring the Treg/Th17 balance in the tumor microenvironment, rather than the direct pro-apoptotic effect on tumor cells.

### 5.5. Neuroprotective Effects

The literature review indicates that in rat models of Parkinson’s disease (PD), DNLA ameliorates 6-hydroxydopamine-induced loss of dopaminergic neurons in the substantia nigra, suggesting their potential as a therapeutic candidate for PD [59]. Alzheimer’s disease (AD), another neurodegenerative disorder, has been studied using LPS-induced hippocampal neuronal injury models. Li demonstrated that DNLA attenuates neuronal damage and cognitive impairment by suppressing NLRP3 inflammasome activation, reducing pro-inflammatory cytokine release (IL-18 and IL-1β), and inhibiting the expression of pyroptosis executor GSDMD-N [60]. This mechanism highlights DNLA’s role in modulating neuroinflammation and pyroptosis, offering a novel strategy for AD intervention. Among the alkaloids, dendrobine-type sesquiterpenoids—including dendrobine, dendrobine-N-oxide, nobilonine, dendroxine, 6-hydroxy-nobilonine, and 3-hydroxy-2-oxodendrobine—play a dominant role in neuroprotection. These compounds act through multi-target mechanisms: inhibiting neuroinflammation, reducing β-amyloid accumulation, enhancing neurotrophic factor expression, suppressing tau hyperphosphorylation, and counteracting neuronal apoptosis, underscoring their potential in treating neurodegenerative diseases [61].

Although dendrobine exhibits prominent neuroprotective, anti-inflammatory and antioxidant properties, it should be noted that dendrobine is classified as a picrotoxane-type sesquiterpenoid alkaloid and shares high structural similarity with the well-known neurotoxin picrotoxinin [62].

Both compounds possess a conserved picrotoxane skeleton, which endows dendrobine with potential toxic risks such as central nervous system depression, convulsion and respiratory inhibition at excessive doses. Therefore, the pharmacological profile of dendrobine should not be only summarized based on its beneficial neuroprotective effects; its structural homology with neurotoxin and corresponding potential safety hazards should also be comprehensively discussed. Currently, there is a lack of quantitative comparative studies between dendrobine and picrotoxinin, suggesting that further systematic toxicological comparative experiments are still needed for the safety evaluation of these structural analogs.

### 5.6. Toxicity and Safety

A large number of studies have demonstrated the positive effects of dendrobine; however, its safety and toxicity cannot be ignored.

DNLA is mainly metabolized in the liver by the cytochrome P450 enzyme system, among which CYP3A4, CYP2C19 and CYP2D6 are the core isoenzymes involved. In vitro human liver microsome experiments showed that DNLA could significantly inhibit the activities of the above three isoenzymes, with IC_50_ values of 12.72, 10.84 and 15.47 µM, respectively. Among them, the inhibition on CYP3A4 was non-competitive and time-dependent (Ki = 6.41 µM, KI = 2.541 µM^−1^, Kinact = 0.0452 min^−1^), while the inhibition on CYP2C19 and CYP2D6 was competitive (Ki = 5.22, Ki = 7.78 µM) with no time dependence [63]. This inhibitory effect suggests that DNLA is prone to interact with drugs or herbal medicines metabolized by CYP450, delay the clearance of exogenous substances, and increase the burden of liver metabolism.

At therapeutic doses, DNLA has a definite protective effect against acute liver injury [64]. In vivo mouse experiments showed that 20 mg/kg dendrobine could significantly reduce ALT and AST levels and improve pathological damage of liver tissues. In vitro AML-12 hepatocyte experiments confirmed that DNLA exerted a concentration dependent protective effect, with weak protection at 3.5 ng/mL and better efficacy at 35 ng/mL [65].

Nevertheless, excessive dose or long-term overuse will eliminate the hepatoprotective effect of DNLA and turn into a risk of liver injury. High-dose DNLA can inhibit the activities of antioxidant enzymes such as SOD, CAT and GSH-Px, increase ROS levels, trigger the mitochondrial apoptotic pathway, and induce oxidative stress and inflammatory damage in hepatocytes. Meanwhile, its inhibition on CYP450 isoenzymes will further amplify liver damage, aggravate the toxicity of itself and combined drugs, and cause transient elevation of liver enzymes and mitochondrial dysfunction. Long-term overuse may also lead to adverse neurological reactions such as dizziness, fatigue and limb numbness, and disturb the normal metabolic homeostasis of liver and kidney [66,67].

Clinical and animal toxicological data further support the dose-related risk. Long-term overdose of *Dendrobium* compound preparations (>30 g/kg) can cause reversible damage to liver and kidney function; common clinical adverse reactions include gastrointestinal discomfort and allergy. In addition, the potential neurotoxicity caused by the structural similarity of DNLA and the high-dose toxicity of preparations cannot be ignored. In conclusion, DNLA possesses definite pharmacological activities such as hepatoprotection and anti-inflammation at therapeutic doses, but it has a narrow therapeutic window. Strict dose control, liver function monitoring and surveillance of CYP450-related drug interactions are required in clinical application, and high-dose and long-term abuse should be avoided.

## 6. Conclusions

As the core representative active ingredient in the DNLA of *D. nobile*, dendrobine possesses definite and diverse pharmacological properties, holding great application prospects in hypoglycemic, anti-inflammatory, anti-aging and neuroprotective research. However, relevant safety assessment and toxicological studies remain insufficient, and substantial efforts are still required to promote its clinical translation. This review systematically elaborates the structural features and tissue distribution patterns of over twenty dendrobine-type alkaloids, offering valuable references for fundamental research, exploitation and utilization of these compounds.

In terms of biosynthesis, the MVA and MEP precursor pathways, together with key enzymes including FPPS, TPS, CYP450, transaminases and methyltransferases, form the core molecular foundation for dendrobine biosynthesis and post-modification. Synthetic biology and metabolic engineering serve as vital strategies to alleviate the shortage of natural resources, elevate dendrobine synthetic efficiency, and achieve its efficient and stable production. Currently, mainstream preparation approaches, including direct extraction, chemical synthesis and microbial biosynthesis, all have distinct pros and cons, which necessitate further optimization targeting key targets, rate-limiting enzymes and metabolic flux regulation.

Overall, future research should focus on mining and functional verification of key synthetic genes, optimizing chassis cells and fermentation processes to construct high-yield cell factories. Combined with standardized cultivation management and inducer application, multi-strategy synergistic regulation can effectively facilitate efficient biosynthesis and accumulation of dendrobine. In conclusion, alkaloids from *D. nobile* possess remarkable phytochemical research significance and promising clinical transformation potential. In-depth exploration of their structure–activity relationships, biosynthetic regulatory mechanisms, metabolic engineering modification strategies, and large-scale preparation systems can lay a solid scientific foundation for their application in innovative drug development, functional product research, and chronic disease clinical intervention.

## Data Availability

No new data were created or analyzed in this study. Data sharing is not applicable to this article.
